# Crystal structure and cation-anion interactions of potassium (Difluoromethanesulfonyl) (trifluorome thanesulfonyl)imide

**DOI:** 10.3389/fchem.2023.1191394

**Published:** 2023-07-12

**Authors:** Eduardo Sánchez-Diez, Lorena Garcia, Oier Arcelus, Lixin Qiao, Leire San Felices, Javier Carrasco, Michel Armand, Maria Martínez-Ibañez, Heng Zhang

**Affiliations:** ^1^ Centre for Cooperative Research on Alternative Energies (CIC EnergiGUNE), Basque Research and Technology Alliance (BRTA), Vitoria-Gasteiz, Spain; ^2^ Servicios Generales de Investigación SGIker, Facultad de Ciencia y Tecnología, Universidad del País Vasco, UPV/EHU, Bilbao, Spain; ^3^ IKERBASQUE, Basque Foundation for Science, Bilbao, Spain; ^4^ Key Laboratory of Material Chemistry for Energy Conversion and Storage (Ministry of Education), School of Chemistry and Chemical Engineering Huazhong, University of Science and Technology, Wuhan, China

**Keywords:** Hydrogen-containing sulfonimide anion, (Difluoromethanesulfonyl)(trifluoromethyanesulfonyl)imide, Crystal structure, Cation-anion interaction, Dihedral angle

## Abstract

Sulfonimide salts are of great interest for battery use thanks to their special properties including sufficient superior chemical/thermal stabilities, structural flexibility, *etc.* In particular, the hydrogen-containing sulfonimide (difluoromethanesulfonyl)(trifluoromethanesulfonyl)imide anion {[N(SO_2_CF_2_H) (SO_2_CF_3_)]^−^, DFTFSI^−^}, stands out owing to its suppressed anion mobility and superior electrochemical properties. We herein report the structural analyses of potassium (difluoromethanesulfonyl)(trifluoromethanesulfonyl)imide {K [N(SO_2_CF_2_H) (SO_2_CF_3_)], KDFTFSI} by virtue of single crystal X-ray diffraction and computational approaches. Our results reveal that KDFTFSI crystallizes in a orthorhombic cell (space group: Pbcn) comprising of cationic and anionic layers, which is similar to the conventional sulfonimide salt, potassium bis(trifluoromethanesulfonyl)imide {K [N(SO_2_CF_3_)_2_], KTFSI}. Gas-phase density functional theory calculations show that the conversion from *trans* to *cis* DFTFSI^−^ anions is hindered due to the presence of stabilizing intramolecular H-bonding interactions in the *trans* conformer; yet interaction with K^+^ substantially minimizes the energy difference between the two conformers due to the formation of strong tridentate K^+^ coordination with oxygen atoms in the *cis* KDFTFSI. This work is anticipated to provide further understanding on the structure-property relations of hydrogenated sulfonimide anions, and thus inspire the structural design of new anions for battery research.

## 1 Introduction

Perfluoroalkyl sulfonimide anions are a family of ions containing sulfonimide anionic center [–SO_2_–N^(−)^–SO_2_–] and fluoroalkyl chains (R_F_, e.g., C_*x*_F_2*x*+1_, C_*x*_F_*y*_H_*z*_), in which the former conjugated unit allows sufficient delocalization of negative charges over four oxygen atoms and one nitrogen atom, while the strong electronegativities of the terminal fluorinated groups further decrease the negative charge densities on nitrogen/oxygen atoms (Linert et al., 2002; Zhang et al., 2017; Zhang et al., 2023). The extremely weak coordinating ability of these perfluoroalkyl sulfonimide anions allows ready access to a variety of inorganic (M^+^ = Li^+^, Na^+^, K^+^, Rb^+^, Cs^+^, *etc.*) and organic (e.g., NR_1_R_2_R_3_R_4_^+^) salts with low melting points and superb chemical/thermal stabilities, being of great interest for electrochemical applications (e.g., batteries, capacitors, electrochromic devices, *etc.*) (Bonhôte et al., 1996; MacFarlane et al., 1999; Tokuda et al., 2006; Tsunashima and Sugiya, 2007; Armand et al., 2009; Eshetu et al., 2016; Oteo et al., 2019).

The bis(trifluoromethanesulfonyl)imide anion {[N(SO_2_CF_3_)_2_]^−^, TFSI^−^}, first suggested as countercharge of electrolyte salts by Armand and Moursli (1983); Zhang and Armand (2021), has aroused significant attention in battery community, owing to their high thermal and electrochemical stabilities (>5.0 V vs. Li/Li^+^) and high ionic conductivities in aprotic solvents. Presently, lithium bis(trifluoromethanesulfonyl)imide has been widely utilized as main conducting salt for building highly Li-ion conductive polymer electrolytes, leading to the commercialization of solid-state lithium metal batteries by Bolloré group (Zhang et al., 2020; Zhang and Armand, 2021). Analogously, the TFSI-based ionic liquids are also highly relevant in electrochemical energy storage applications due to their low viscosities, high ionic conductivities, low melting points and high stability, both thermal, as compared to conventional liquid electrolytes, and electrochemical, e.g., high anodic stability (Armand et al., 2009; Eshetu et al., 2016). Unfortunately, the application of LiTFSI in non-aqueous liquid electrolyte is severely hindered by its corrosive nature towards aluminum current collector above ca. 3.8 V vs. Li/Li^+^ (Krause et al., 1997; Oteo et al., 2019; Zhang et al., 2019; Qiao et al., 2022).

Recently a novel hydrogen-containing sulfonimide salt, (difluoromethanesulfonyl)(trifluoromethanesulfonyl)imide {Li [N(SO_2_CF_2_H)(SO_2_CF_3_)], LiDFTFSI} was reported by our group (Oteo et al., 2019; Zhang et al., 2019; Qiao et al., 2022). This salt could be readily obtained with cost-effective synthetic approaches, and shows high anodic stability and good compatibility with aluminum current collector (>4.2 V vs. Li/Li^+^) while it presents high chemical stability. The hydrogen atom plays a critical role as it is believed to form LiH, with Li-ion conductive nature, which regulates the properties of the solid-electrolyte-interphase (SEI) layer formed on the lithium metal (Li°) electrode (Qiao et al., 2022). In the case of solid polymer electrolytes, the use of LiDFTFSI lead to an increase of the lithium-ion conductivity by anion-anion and anion-polymer interactions via H-bonding, limiting the migration of anionic species (Lee et al., 1988; Oteo et al., 2019; Zhang et al., 2019). Thus, LiDFTSI stands as a safe option for both liquid and solid electrolytes and circumvents the problems that classic LiTFSI exhibits (Oteo et al., 2019; Zhang et al., 2019; Qiao et al., 2022).

The effect of C-H···F like hydrogen bond interactions on packing of crystalline structures has been previously identified (Becke, 1993). Those, in addition to other weak interactions such as fluorine-metal and fluorine-fluorine interactions, may have a significant contribution in sulfonimide salts with a extensive delocalization of the negative charge. In this sense, crystal structures of LiTFSI, NaTFSI, KTFSI and CsTFSI (Matsumoto et al., 2015) have been reported describing their layered structured with prevalent metal-oxygen and metal-nitrogen interactions. Detailed structural data obtained by X-ray diffraction may serve to identify possible hydrogen bonding interactions on DFTFSI salts as anticipated in electrolyte analysis studies.

Currently, extensive efforts have been devoted to a deeper understanding or evaluation of the properties of TFSI-based electrolytes while little is known about DFTFSI and its full potential. Indeed, it has been observed that cesium (difluoromethanesulfonyl)(trifluoromethanesulfonyl)imide {Cs [N(SO_2_CF_2_H)(SO_2_CF_3_)], CsDFTFSI} has a very low melting point (72°C) (Oteo et al., 2019), becoming a potential candidate for a low-temperature molten salt electrolytes. In this work, the crystal structure of KDFTFSI determined by single-crystal X-ray diffraction is reported and the results are discussed and compared with previously reported structure of KTFSI (Xue et al., 2002). It is important to highlight that potassium salts are much less sensitive toward humidity, allowing the successful preparation of single crystals without any contamination of solvents, as generally observed for lithium and sodium salts. Coordination environment of potassium atoms and relative disposition of fluoroalkyl terminals are comprehensively studied. Computational studies of the bare anion, lithium and potassium salts are also included, to shed some light on the preferential conformation of the DFTFSI^−^ anion. The knowledge accumulated with the hydrogenated sulfonimide salts is likely to be transferable among different electrolytes ranging from lithium/sodium to potassium-based systems (Zhang et al., 2023).

## 2 Materials and methods

### 2.1 Synthesis and characterization

#### 2.1.1 Materials

Potassium hydroxide (KOH, Scharlab), acetone (99%, Scharlab), difluoromethanesulfonyl chloride (CF_2_HSO_2_Cl, Manchester Organics Limited), 1-methylimidazole (Sigma-Aldrich), acetonitrile (anhydrous, 99.9%, Thermo-Scientific), hydrochloric acid (HCl, 37%, Scharlab), *tert*-butyl methyl ether (MTBE, anhydrous, 99.8%, Sigma-Aldrich), potassium hydrogen carbonate (KHCO_3_, Thermo-Scientific), and deuterated acetone (acetone-*d*_6_, Eurisotop, 99.8% D) were used as purchased. Trifluoromethanesulfonamide (CF_3_SO_2_NH_2_) was a generous gift from Solvay.

#### 2.1.2 Characterization methods

Structure characterizations were carried out using a liquid nuclear magnetic resonance (NMR) spectroscopy, attenuated total reflectance-Fourier-transform infrared spectroscopy (ATR–FTIR), and the ultra-high performance liquid chromatography-quadrupole time-of-flight mass spectrometry (UPLC/Q–TOF–MS). NMR spectra were acquired on a Bruker 300 Ultrashield (300 MHz for ^1^H, 75.5 MHz for ^13^C and 282 MHz for ^19^F). Chemical shifts (δ) are reported in ppm relative to residual solvent signals (i.e., acetone, 2.05 ppm for ^1^H-NMR; (CD_3_)_2_CO, 29.84 ppm for ^13^C-NMR) or internal reference (CCl_3_F). The following abbreviations are used to indicate the multiplicity in ^1^H–NMR, ^13^C–NMR, and ^19^F–NMR spectra: *s*, singlet; *d*, doublet; *t*, triplet; *q*, quartet. ^13^C–NMR spectra were acquired on a broad band decoupled mode using distortionless enhancement by polarization transfer (DEPT) experiments for assigning different type of carbon environment. These measurements were carried out using deuterated acetone (CD_3_)_2_CO (99.8%) as solvent at 25°C. ATR-FTIR spectra were collected using an Agilent Technologies Cary 630 spectrometer setup inside an Argon filled glove box. The spectrum with a spectral range spanning 4,000–650 cm^−1^ and a resolution of 2 cm^–1^ was averaged from 64 scans. The following abbreviations are used to rate the intensity of the bands in IR spectra: s, strong; m, medium; w, weak. UPLC/Q–TOF–MS experiments consisted in a chromatographic separation in an ultrahigh performance liquid chromatograph (UPLC, Acquity system) coupled to a high-resolution mass spectrometer [Synapt G2 HDMS, time of flight analyser (TOF)] by an electrospray ionization (ESI).

#### 2.1.3 Synthesis of KDFTFSI

(Difluoromethanesulfonyl)(trifluoromethanesulfonyl)imide in acid form {H [N(SO_2_CF_2_H) (SO_2_CF_3_)], HDFTFSI} was prepared from the reaction of potassium trifluoromethanesulfonamide (CF_3_SO_2_NHK) with difluoromethanesulfonyl chloride (CF_2_HSO_2_Cl) in 1:1 M ratio in acetonitrile heating to reflux for 48 h, following the previously reported procedures (Oteo et al., 2019). The potassium salt was obtained by the neutralization of the acid form employing potassium hydrogen carbonate in ethanol stirring at room temperature for 16 h. After that time, the remaining solid was filtered, solvents were evaporated and the salt was crystallized from ethanol/toluene (1/1, vol/vol) giving potassium (difluoromethanesulfonyl)(trifluoromethanesulfonyl)imide {K [N(SO_2_CF_2_H) (SO_2_CF_3_)], KDFTFSI} as a white powder. ^1^H NMR (300 MHz, acetone-*d*_6_, TMS, ppm): δ = 6.37 (*t*, *J* = 54.3 Hz, 1H). ^13^C NMR (75 MHz, acetone-*d*_6_, TMS, ppm): δ = 121.06 (*q*, *J* = 321.6 Hz), 114.84 (*t*, *J* = 277.5 Hz). ^19^F NMR (282 MHz, acetone-*d*_6_, CCl_3_F, ppm): δ = −79.50 (s, 3F), −124.99 (d, *J* = 53.3 Hz, 2F). Infrared [frequency/cm^−1^ (relative intensity, w: weak, m: middle, s: strong)]: 1,325 (m), 1,195 (m), 1,172 (m), 1,118 (s), 1,060 (s), 801 (w), 777 (w), 743 (m). ESI^–^-MS (m/z) for KDFTFSI: 261.9267 (theoretical). Found: 261.9272.

### 2.2 X-ray diffraction analysis

Crystallographic data including experiment conditions and refinement results are given in Table 1. KTFSI data was obtained from literature (Xue et al., 2002). The data for KDFTFSI was recorded at reduced temperature (150 K). A suitable crystal was selected and mounted on a SuperNova, Single Source at 53 mm from detector, Atlas diffractometer equipped with monochromated Cu *K*α radiation (λ = 1.54184). Standard reflections were measured periodically with no significant variations in intensity during data acquisition. High-angle reflections allowed to precisely determine the cell dimensions by refinement of the setting angles. Lorentz polarization corrections were applied. Neutral atom scattering factors, coefficients of anomalous dispersion and absorption coefficients were taken from International Tables of X-ray Crystallography” (Moss, 1975). All atoms were anisotropically refined with the exception of H atoms which were refined isotropically on the assumption of a ride-on model with their Uiso fixed to 1.2 × Ueq of the C atoms. Using Olex2 (Dolomanov et al., 2009), the structure was solved with the ShelXT (Sheldrick, 2015) structure solution program using Intrinsic Phasing and refined with the ShelXL (Sheldrick, 2015) refinement package using Least Squares minimization (on *F* ^2^).

**TABLE 1:** Crystallographic data of KDFTFSI and KTFSI salts.

Compound	KDFTFSI	KTFSI ^a^
Formula	K [(CF_3_SO_2_)(CHF_2_SO_2_)N]	K[(CF_3_SO_2_)_2_N]
Molecular weight (g mol^−1^)	301.26	319.25
Crystal size (nm^3^)	0.576 × 0.373 × 0.118	0.3 × 0.3 × 0.3
Crystal system	orthorhombic	orthorhombic
Space group (No.)	Pbcn (No. 60)	Pbcn (No. 60)
a (Å)	21.8280 (6)	22.346 (7)
b (Å)	13.3610 (3)	13.541 (3)
c (Å)	12.4932 (4)	12.860 (3)
α (°)	90.0	90.0
β (°)	90.0	90.0
γ (°)	90.0	90.0
Volume (Å^3^)	3,643.59 (18)	3,891 (1)
Z	16	16
Diffractometer	Atlas	Siemens R3mV
Temperature (K)	150	295 ± 1
2*θ* range for data collection (°)	7.758–137.972	1.76–22.56 (*θ* range)
*µ* (mm^−1^)	10.318	1.07
Refls meas	25,930	2,560
Refls uniq (Rmerg)	3,391	2,560 (0.0)
R1	0.0754	0.0478 (0.0996)
ω R2	0.2006	0.0884 (0.0935)

^a^ The crystallographic data of KTFSI, is obtained from (Xue et al., 2002).

Complete list of bonding parameters, atom coordinates, anisotropic displacements and atomic occupancy are given in Supplementary Tables S1‒S5. Selected bond lengths and angles are summarized in Table 2. Crystallographic data was deposited with the Cambridge Crystallographic Data Centre (Moss, 1975) CCDC 2242830.

**TABLE 2:** Bonding parameters of KDFTFSI and KTFSI salts.

Compound	KDFTFSI	KTFSI ^a^
N‒S (Å)	1.573(5), 1.581(6)	1.570(6), 1.571(6)
	1.580(5), 1.562(6)	1.575(6), 1.581(6)
S‒O (Å)	1.433(5), 1.435(5)	1.431(5), 1.440(5)
	1.439(5), 1.435(5)	1.437(5), 1.438(5)
	1.426(5), 1.436(5)	1.439(5), 1.425(5)
	1.436(5),1.429(5)	1.438(5), 1.438(5)
S‒C (Å)	1.807(8), 1.719(7)	1.839(9), 1.84(1)
	1.726(7), 1.826(7)	1.84(1), 1.84(1)
S‒N‒S (°)	128.0(4), 125.7(3)	128.2(4), 126.3(4)
N‒S‒O (°)	116.5(3), 109.5(3)	116.4(3), 109.8(4)
	106.4(3), 116.3(3)	107.2(3), 116.1(3)
	106.8(3), 115.5(3)	108.1(4), 116.9(3)
	117.0(3), 108.7(3)	117.4(3), 105.8(4)
N‒S‒C (°)	104.4(3), 106.0(3)	104.0(4), 105.1(4)
	105.9(3), 103.7(3)	104.2(5), 105.9(4)
O‒S‒O (°)	116.1(3), 117.3(3)	116.0(3), 116.7(4)
	117.2(3), 116.9(3)	116.3(4), 117.0(4)
O‒S‒C (°)	105.0(3), 103.5(3)	105.4(4), 103.5(4)
	104.8(4), 104.9(4)	104.7(5), 105.8(5)
	105.0(3), 105.4(3)	104.6(5), 105.3(5)
	105.4(3), 103.2(3)	106.1(5), 103.2(5)
M···O (Å) avg	2.864(5)	2.9(2)
M···O (Å) range	2.687(5) −3.338(6)	2.697(6) −3.343(6)
M···N (Å)	3.295(6), 3.365(5)	3.405, 3.413
C‒S···S‒C (|°|)	17.1, 20.0	13.2, 9.4

^a^ The data of KTFSI, is obtained from (Xue et al., 2002).

### 2.3 Computation

The density functional theory (DFT) calculations of the isolated anions and salts were performed using the M08-SO exchange-correlation functional (Zhao and Truhlar, 2008) and the “tier2” standard basis set with “tight” settings, as implemented in the numeric atom-centered basis all-electron code FHI-aims (Blum et al., 2009; Havu et al., 2009) for K, C, N, O, F, S, and H atoms. The initial structure of the TFSI^−^ anion was first extracted from our previous work (Zhang et al., 2018), and the energy differences between *cis* and *trans* configurations were well reproduced. Then, the molecular editor Avogadro (Hanwell et al., 2012) was used to generate a range of initial structures for the DFTFSI^−^ anions. We considered five conformers of the DFTFSI^−^ anions for each *cis* and *trans* configuration, representing the rotational degrees of freedom of the ‒CF_2_H group. After geometry optimization with FHI-aims, we considered the lowest-energy *cis* and *trans* configurations for both TFSI^−^ and DFTFSI^−^ anions to calculate their interaction with a K^+^ cation. We generated 10 and 15 different configurations of K^+^ attached to different sites of the TFSI^−^ and DFTFSI^−^ anions, respectively, and relaxed them. All geometries were optimized with a residual force threshold of 1 × 10^−2^ eV Å^−1^ using the trust radius method enhanced version of the Broyden-Fletcher-Goldfarb-Shanno (BFGS) optimization algorithm (Nocedal and Wright, 2006). The convergence of electron density and the total energy of the system were set to 1 × 10^−4^ e and 1 × 10^−6^ eV, respectively.

The effect of the solvent was included in the relaxed geometries using the multipole moment expansion (MPE) model (Filser et al., 2022), which allows for an efficient way of including coarse solvation effects in full potential all-electrode codes such as FHI-aims. For the solvent, a dynamic cavity is used, which is constructed as an iso-density surface of the electron density of the solute at each self-consistent field cycle with an iso-value of 0.125 e Å^−3^. Lacking a clear estimate of the value of the dielectric constant (ε) for both KTFSI and KDFTFSI crystals, we calculated the energy differences between the *cis* and *trans* configurations of the most stable TFSI^−^, DFTFSI^−^, KTFSI and KDFTFSI molecules in the range ε = 1–60.

We also constructed periodic models for the KTFSI and KDFTFSI crystals. The structural models for the crystals with *cis* conforming anions of both KTFSI and KDFTFSI were extracted from the single crystal diffraction results presented in this work and previous works (Xue et al., 2002). Due to the lack of experimental data for potassium salts with *trans* conforming anions, we used the lithium salt models originally reported (Nowinski et al., 1994), where Li ions were substituted by K ions. The disorder in the H and F positions for the experimental KDFTFSI structure required building ordered models that were too large to be handled by DFT calculations. Thus, we approximated these structures by manually ensuring that each anion contained only one H atom. Furthermore, we did not explore the configurational phase space for all possible H–F orderings. However, we do not expect this approximation to have a large effect on the overall stability of the KDFTFSI structural models.

The structural relaxations of the crystal structures were again performed with FHI-aims. First, fast pre-relaxations were performed using “tier1” basis sets and “light” numerical settings. Here, the atomic positions, lattice parameters, and cell angles were optimized with a residual force threshold of 1 × 10^−2^ eV Å^−1^ using the BFGS algorithm. Then, a tighter optimization was performed, using a “tier2” basis and “light” numerical settings, until the forces again reached the threshold 1 × 10^−2^ eV Å^−1^. All structural relaxations were performed with a 2 × 2 × 2 (2 × 4 × 2) k-point grid for the structures with *cis* (*trans*) conforming anions. Lastly, additional single step electronic structure calculations were performed by using “tier2” basis and “tight” numerical settings on the optimized structures, using a 4 × 4 × 4 (4 × 8 × 4) k-point grid for the structures with *cis* (*trans*) conforming anions. These settings ensure a tight convergence of the self-consistent energies. Regarding the exchange-correlation functional, we used the standard Perdew–Burke–Ernzerhof (PBE) functional as implemented in FHI-aims for periodic calculations (Perdew et al., 1996), including van der Walls dispersion effects using the Hirshfeld partitioning of the electron density, as described by Tkatchenko and Scheffler (2009).

All the structures and information about the simulation are made available publicly through the NOMAD repository (https://doi.org/10.17172/NOMAD/2023.05.30-1).

## 3 Results and discussion

Crystallographic data on measured monocrystal of KDFTFSI is presented. Details on the conditions and refinement are given in Table 1. Further details can be found in Supplementary Tables S1‒S6.

Bis(perfluoroalkylsulfonyl)imides may present both *trans* and *cis* conformations according to their relative positions of the perfluoroalkyl groups with respect to the S‒N‒S plane. While *trans* conformation is typically thermodynamically favoured (Johansson et al., 1998) *cis* conformation is commonly observed in crystalline structure of alkali metal salts (Matsumoto et al., 2013). The cation-anion interactions diminish the energy barrier between *cisoid* and *transoid* forms as the chelation of the anion becomes stronger. The potassium salt of (difluoromethanesulfonyl)(trifluoromethanesulfonyl)imide (KDFTFSI) also presents a *cisoid* conformation. As seen in Table 1, both KDFTFSI and KTFSI compounds present an orthorhombic structure and similar cell dimensions.

**FIGURE 1:**
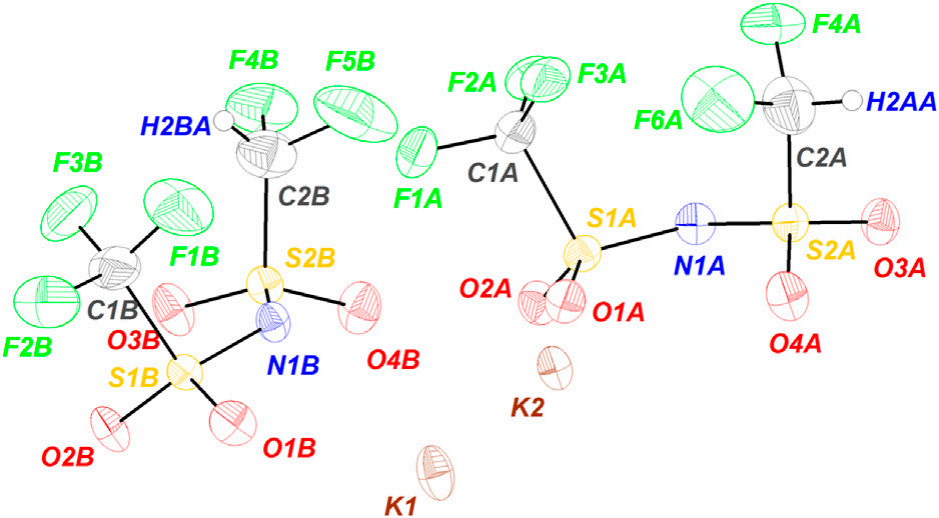
ORTEP diagram of the asymmetric unit of KDFTFSI. The represented structure corresponds to the highest occupancy/portion of the disorder. Ellipsoids are shown at 50% probability.

The ORTEP diagram of the asymmetric unit is shown in Figure 1 and comprises two ion pairs. It should be noted that the representative diagram shows the structure with the highest occupancy as there is certain positional disorders in the C‒H and C‒F of the CF_2_H group. While one of the C‒F in this group is fixed, the other two positions have a similar population distribution. The atomic occupancy for each H atom is included in the (Supplementary Table S5). Larger displacement parameters are observed for the F atoms in the terminal CF_3_ and CF_2_H groups than those of the other atoms (see, Supplementary Table S3), this may relate to some rotational freedom of the CF_3_ and CF_2_H group around the S‒C bond. Certainly, this phenomenon also identified in KTFSI (Matsumoto et al., 2015) is more clearly observed in KDFTFSI due to the substitution of a fluorine atom in the CF_3_ group.

As seen in Figure 1, the diagram shows two ion pairs with the potassium atoms located in the center of the asymmetric unit, the hydrophilic domain of the anion points toward the cations while the hydrophobic fluoroalkylated groups stand away from the potassium atoms.

Attending to bond lengths and angles, the intramolecular geometry of KDFTFSI is similar to that of KTFSI. Table 2 includes a list of bonding parameters for KDFTFSI, and the data of KTFSI is provided for comparison. The complete list of bonding parameters for KDFTFSI is included in Supplementary Table S1. Thus, bond length range: N‒S, 1.562(6)‒1.581(6) Å; S‒O, 1.426(5)-1.439(5) Å; S‒C, 1.719(7)‒1.826(7) Å; C‒F, 1.278(13)‒1.3888(10) Å; and a bond angle range: S‒N‒S, 125.7(3)‒128.0(4)°; N‒S‒O, 106.4(3)‒117.0(3)°; N‒S‒C, 103.7(3)‒106.0(3)°; O‒S‒O, 116.1(3)‒117.3(3)°; O‒S‒C, 103.5(3)‒105.4(3)°; S‒C‒F, 107.8(5)‒118.6(5)°; F‒C‒F, 98.8(7)‒111.2(7)°. The DFTFSI^−^ anions present a *cis* conformation with a C‒S···S‒C torsion angle of 20° (B) and 17° (A), respectively.

**FIGURE 2:**
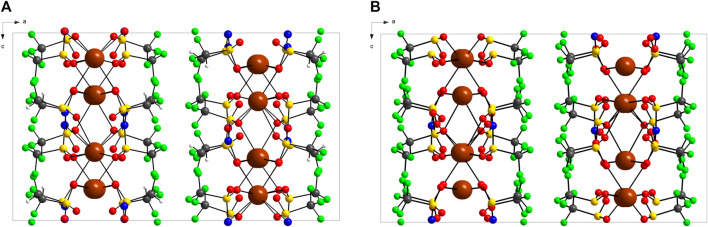
Packing diagram of **(A)** KDFTFSI and **(B)** KTFSI along the *b* axis. The structure with highest population is represented as for ORTEP. The data of KTFSI is obtained from (Xue et al., 2002).

The crystal packing of KDFTFSI and KTFSI is illustrated in Figure 2. Both salts show a layered structure alternating cationic and anionic layers stack along the *b* axis. The potassium cations are sandwiched by the oxygen atoms in the sulfonyl groups, this structure is promoted by the favoured K···O interactions that push aside the fluorine rich groups (CF_3_, CF_2_H) that adopt a *cis*-conformation leading to the fluorine rich layer. This stacking pattern is present in the crystalline structure of those fluorinated sulfonimide salts (NaTFSI (Matsumoto et al., 2015), KTFSI (Xue et al., 2002), CsTFSI (Xue et al., 2005), Sr(TFSI)_2_ (Zak et al., 1998) with only minor differences in the repeating distance of those layers being attributed to the cation size (Matsumoto et al., 2015) and no major differences between similar sulfonimide anions (TFSI^−^, FSI^−^, and DFTFSI^−^) (Matsumoto et al., 2013). This distance corresponds in orthorhombic structures as KTFSI and KDFTFSI, to approximately half of the value of *a* cell parameter. Thus, a value of 11.276 Å is obtained for KDFTFSI while a value of 11.173 Å is inferred from the cell parameters in KTFSI (Matsumoto et al., 2015). Alternatively, sulfonimide salts with a *transoid* disposition in the crystalline phase, such as LiTFSI (Nowinski et al., 1994) and NaTFSI (Matsumoto et al., 2013) present a relatively simpler packing pattern where cations are simple bridging anion layers.

**FIGURE 3:**
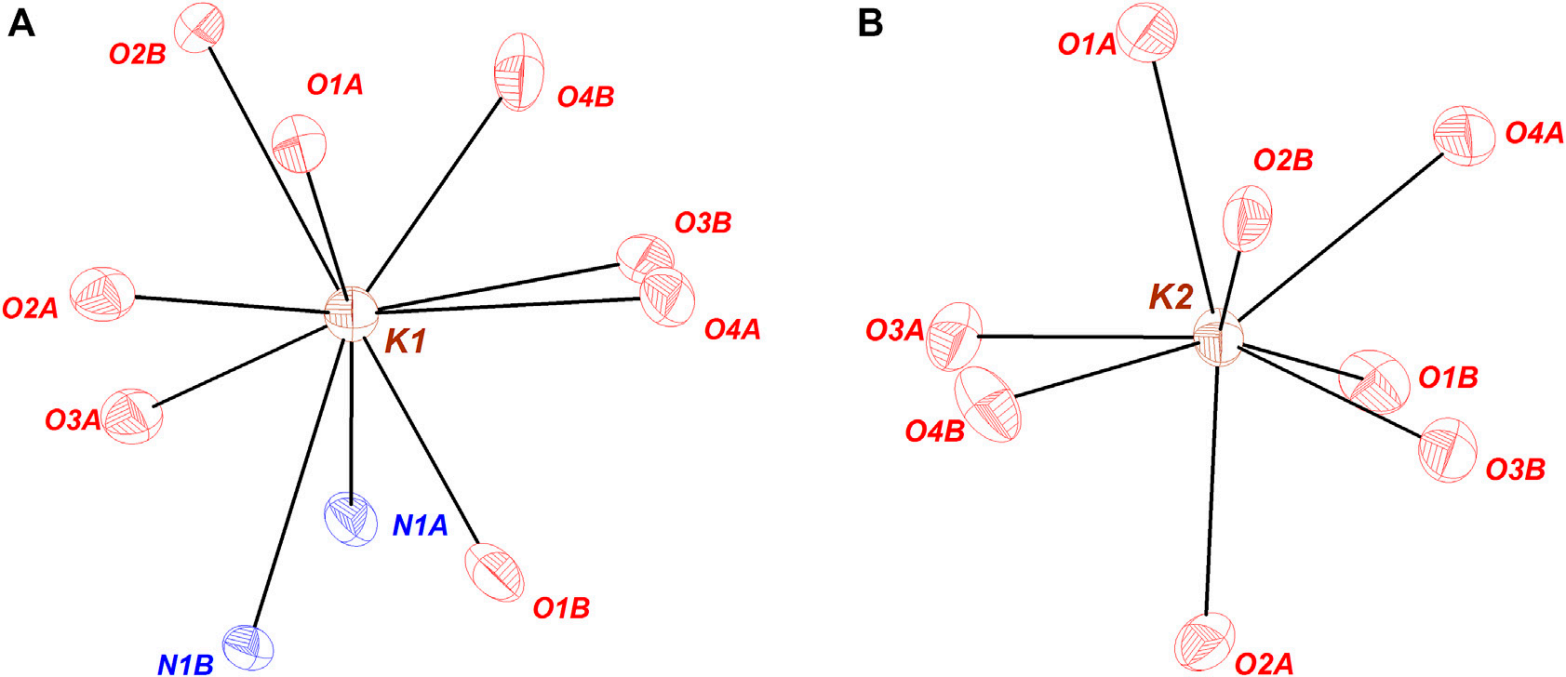
Coordination environment around the crystallographically independent K^+^cations in KDFTFSI. Atoms that do not present x,y,z are listed here: **(A)** N1A, O3A (1.5-x, 0.5-y,-0.5 + z); O2B (1.5-x, 0.5-y,-0.5 + z); O3B, O4B (1.5-x,-0.5 + y, z); O4A (x,-y, 0.5 + z); **(B)** O1A, O4A (1.5-x, 0.5 + y, z); O1B (1.5-x, 0.5-y, 0.5 + z); O2B, O3B (x, 1-y,-0.5 + z); O3A (1.5-x, 0.5-y,-0.5 + z).

The coordination environment of the cations in KDFTFSI is also investigated and compared with the reference KTFSI salt. Figure 3 represents the coordination of each of the potassium atoms identified in both KDFTFSI and KTFSI salts. Potassium cation atoms are mainly coordinated with oxygen atoms from the two sulfonyl groups. Thus, one of the potassium atoms presents primary interactions with four O atoms and secondary interactions with four O atoms and 2 N atoms. The second potassium atom is exclusively coordinated to oxygen atoms through seven primary interactions and one secondary interaction (see Table 3). The relative dispositions adopted by potassium and the O and/or N atoms correspond to pentagonal antiprism and triangular dodecahedron coordination geometries, respectively. K1 atom in KTFSI presents a distorted square antiprism disposition according to previous reports (Xue et al., 2002).

**TABLE 3:** Contact distances (Å), the corresponding bond valences, and bond valence sum (BVS) for KDFTFSI and KTFSI.

Compound	Contact atoms	Contact distance (Å)	Bond valence^a^
KDFTFSI	K1···O	2.746	0.19
	K1···O	2.748	0.19
	K1···O	2.783	0.17
	K1···O	2.840	0.15
	K1···O	2.961	0.11
	K1···O	3.091	0.07
	K1···O	3.192	0.06
	K1···O	3.338	0.04
	K1···N	3.295	0.06
	K1···N	3.365	0.05
			BVS 1.09
	K2···O	2.687	0.22
	K2···O	2.713	0.21
	K2···O	2.727	0.20
	K2···O	2.733	0.20
	K2···O	2.758	0.18
	K2···O	2.791	0.17
	K2···O	2.802	0.16
	K2···O	2.906	0.12
			BVS 1.47
KTFSI^b^	K1···O	2.697	0.22
	K1···O	2.724	0.20
	K1···O	2.726	0.20
	K1···O	2.742	0.19
	K1···O	2.791	0.17
	K1···O	2.848	0.14
	K1···O	2.882	0.13
	K1···O	2.996	0.10
			BVS 1.35
	K2···O	2.800	0.16
	K2···O	2.873	0.13
	K2···O	2.895	0.13
	K2···O	2.959	0.11
	K2···O	3.113	0.07
	K2···O	3.114	0.07
	K2···O	3.204	0.06
	K2···O	3.343	0.04
	K2···N	3.405	0.05
	K2···N	3.413	0.04
			BVS 0.86

^a^ Bond valence was calculated by Eq. 1 where R_0_ (Å) = 2.132 for K···O, and 2.26 for K···N, B = 0.37, and R (Å) corresponds to the experimental contact distance (Brown and Altermatt, 1985).

^b^ The crystallographic data were taken from Xue et al. (2002); Matsumoto et al. (2015).

**FIGURE 4:**
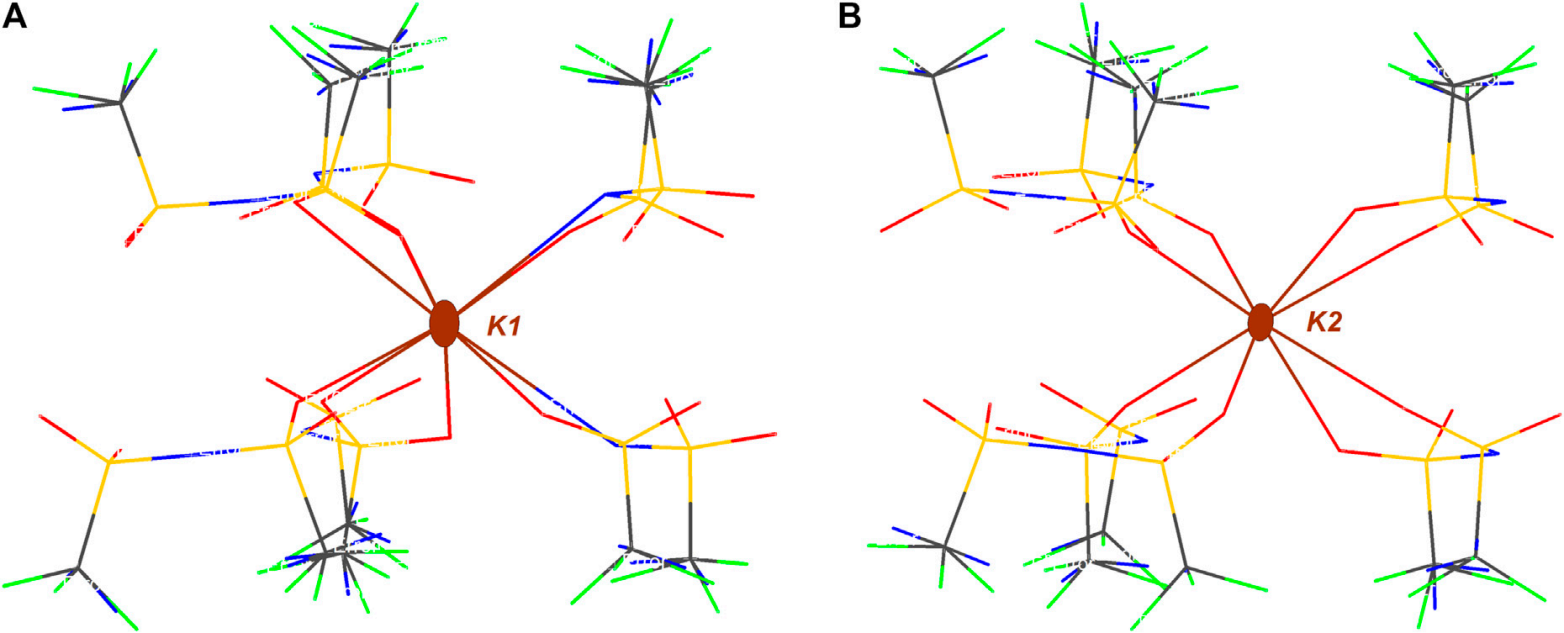
Intermolecular interactions of independent K^+^ atoms in KDFTFSI. **(A)** Environment of K1 coordinated to eight O atoms and two N atomos and **(B)** environment of K2 coordinated to eight O atoms.

As it can be observed in Figure 4, a total of six anions are interacting with K1 cation, four of those coordinate as bidentate ligands forming the corresponding chelates. It is worth mentioning that when anions act as monodentate ligands, the anion is exclusively interacting through oxygen atoms while there is no N-coordinating monodentate anion. K2 cation is also coordinated by six anions, in this case only two of those sulfonimide anions interact in a bidentate fashion.

$$v=\exp\frac{\left[R_{0}-R\right]}{B}$$ (1)

In order to better understand the strength of each of those interactions, the bond valence (ν) and the summatory, defined as bond valence sum (BVS) were determined and therefore quantify their contribution to the coordination state. BVS was calculated for each cation (K1, K2) and bond valence was calculated according to Equation 1: where R_0_ and B are empirically determined parameters that serve to correlate the bond distance and the bond valence for a certain interaction. Those parameters have been tabulated for a variety of atom-atom interactions and are obtained from a systematic analysis of the Inorganic Crystal Structure Database. R_0_ for K···O and K···N atoms is defined as 2.132 and 2.26 Å respectively, B is equal to 0.37, and R is the experimental distance (Brown and Altermatt, 1985). The results of bond valence calculation are summarized in Table 3. Clearly, the values obtained for KDFTFSI show the higher contribution of O over N and also the stronger interaction of oxygen in contact with K1 as compared to those in contact with K2. In addition, it can be observed that the contribution of the so-called secondary interactions cannot be neglected (0.05 vs. 0.19).

In comparison with KTFSI (Matsumoto et al., 2015), potassium atoms in KDFTFSI present a similar coordination environment. Both structures present two types of potassium cations, a K^+^ cation that coordinates with six anions only through K···O interactions and a second K^+^ cation that is also interacting with six anions but exhibits two additional K···N interactions. Thus, despite the differences in their coordination geometries, both KDFTFSI and KTFSI coordinate in a similar way to the anions in terms of the number of anions involved in the coordination as well as in the anion atoms involved in the coordination (Figure 4 and Matsumoto et al., 2015). Different from sodium salts reported in the literature (Matsumoto et al., 2015) the bond valence sums of potassium atoms within the same structure (i.e., K1 vs. K2) in KDFTFSI (1.09, 1.47) and KTFSI (1.35, 0.86) differ significantly. This difference is slightly higher in KTFSI (0.49). Indeed, KDFTFSI presents in general higher bond valence for K···O contacts which may relate to stronger interactions. This phenomenon could be ascribed to the lower dissociation energy/higher delocalization in the TFSI^−^ anion, which could lead to several lower-level interactions of the cation that are not considered within this list of primary and secondary interactions.

**FIGURE 5:**
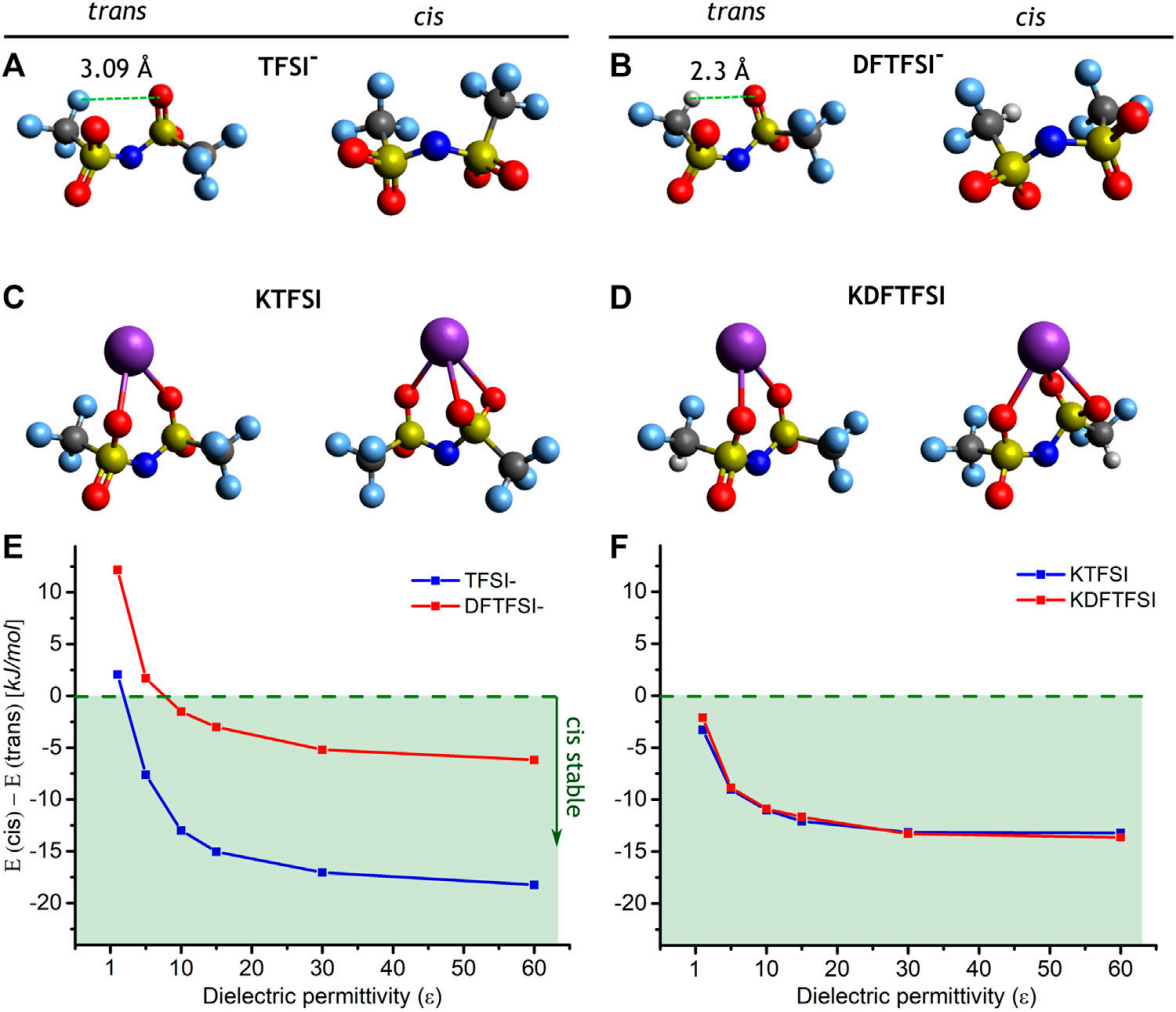
Optimized structures of **(A)** TFSI^−^ and **(B)** DFTFSI^−^ anions, and **(C)** KTFSI and **(D)** KDFTFSI salts, in their *trans* (left) and *cis* (right) conformations. The energy differences between the conformation of **(E)** TFSI^−^ and DFTFSI^−^ anions and **(F)** KTFSI and KDFTFSI ion-pairs, is also given as a function of the dielectric permittivity of the MPE implicit solvent model, the green area indicates the stability of the *cis* conformers. The atoms are represented as white (H), turquoise (F), dark grey (C), yellow (S), blue (N), red (O), and purple (K).

As shown in Table 2, both KTFSI and KDFTFSI present a distorted *cis* conformation. Those torsion angles formed by the fluorinated terminals are slightly higher in KDFTFSI. To gain further insights into the preferential disposition of those anions and the effect of the anion and the cation, we performed a series of DFT calculations to compute the energy difference between the *cis* and *trans* conformations in TFSI^−^ and DFTFSI^−^anions, as well as for the corresponding potassium salts (Figure 5). Our DFT calculations reveal that, in the gas phase, the *trans* conformation is the most stable in both TFSI^−^ and DFTFSI^−^ anions. Specifically, the *trans*-TFSI^−^ (DFTFSI^−^) is 2.1 (12.2) kJ mol^−1^ more stable than the *cis* conformer. This is because of internal atomic distortions of the *trans* conformer that enable an additional H···O interaction (2.3 Å) between the hydrogen atom from the CF_2_H group and the oxygen atom from the opposed sulfonyl group. (Figure 5B). Interestingly, the inclusion of a screening dielectric medium by means of an MPE implicit solvent model flips the stability of *cis* and *trans* cations (Figure 5E), where the *cis* already becomes the preferred conformation at relatively low values of dielectric constants for both TFSI^−^ and DFTFSI^−^ anions. The interactions with alkaline cations in the corresponding salts have a major impact on the stability of the conformers. Previous works have showed that the LiTFSI salt has an increased energy gap, where the *trans* conformer was 5.0 kJ mol^−1^ more stable than its *cis* counterpart (Zhang et al., 2018). In fact, a preferential disposition for the *trans* conformer is also observed in the crystalline structure of LiTFSI (Nowinski et al., 1994; Matsumoto et al., 2015). We note that this gap observed for LiTFSI is larger than the one considering the TFSI^−^ anion alone. Interestingly, the contrary happens when considering the potassium salt in the gas phase, where the *trans*-*cis* energy gap is again flipped to 3.3 kJ mol^−1^ for KTFSI and 2.1 kJ mol^−1^ for KDFTFSI, this time favoring the *cis* conformer. Similar to the case of the isolated anions, the *cis* conformer gets further stabilized by the inclusion of an implicit solvent (Figure 5F), where the *trans-cis* energy gap increases to 13.1 kJ mol^−1^ for KTFSI and 13.6 kJ mol^−1^ for KDFTFSI. This is a consequence of the larger size and polarizability of K^+^ in comparison with Li^+^, which enables a K···O tridentate coordination (Figures 5C, D) in contrast to the bidentate coordination established in the lithium systems. These results reveal two effects that tip the balance between *cis* and *trans* conformations of the potassium salts. On the one hand, the inclusion of an isotropic dielectric medium stabilizes the *cis* conformers of both isolated anions and salts. On the other hand, the inclusion of the K^+^ cation itself further stabilizes the *cis* conformer. This suggests that KTFSI and KDFTFSI ion-pairs will prefer to adopt the *cis* conformation when precipitated from a saturated solution, these preliminary nucleation points would then dictate the patterning for further crystal growth.

**FIGURE 6:**
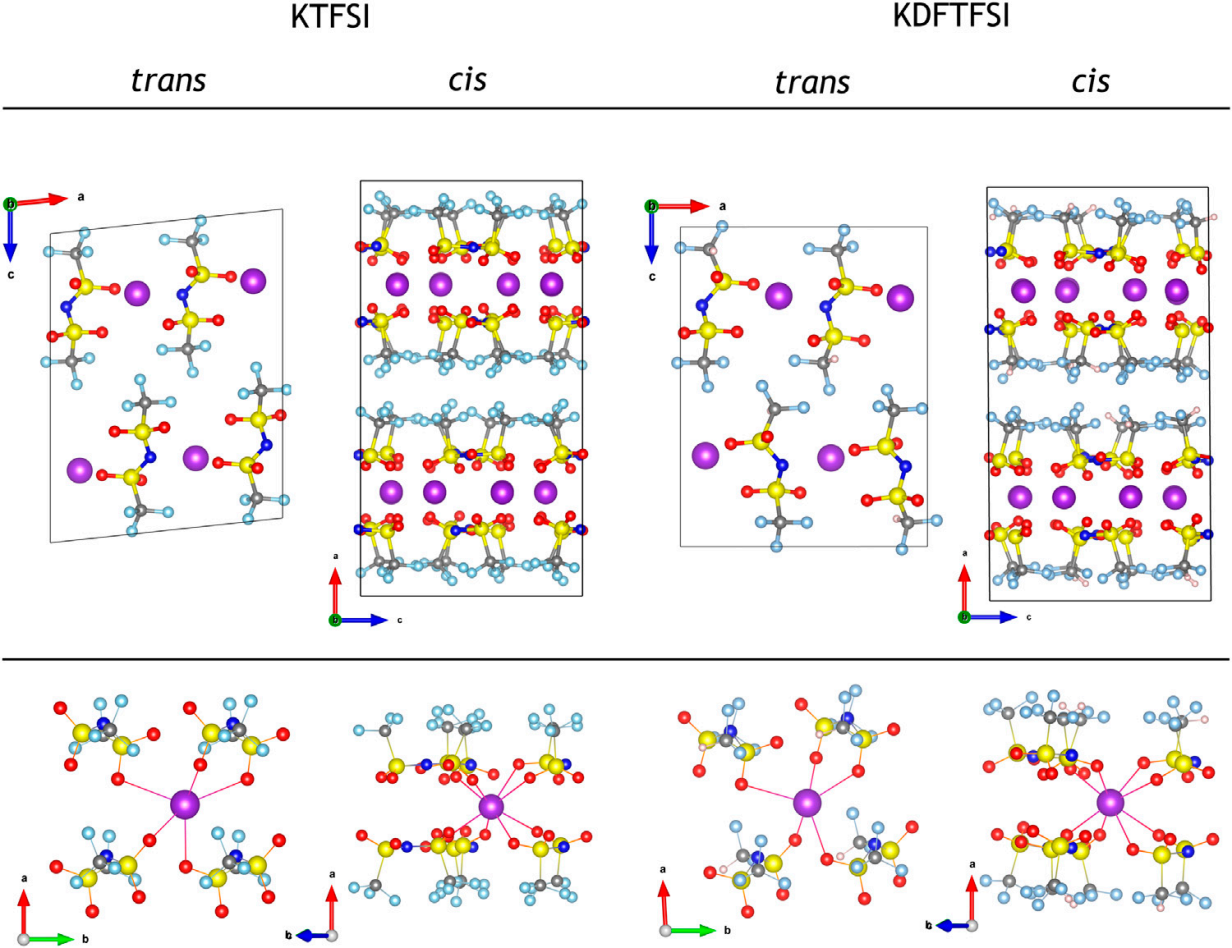
Optimized crystal structures of KTFSI and KDFTFSI salts (top), where the anion units are in *trans* and *cis* conformations, and their corresponding K···O coordination is also highlighted (bottom). The *trans* conforming crystal structures are modeled after the work of Nowinski et al. (1994). The color code is maintained from Figure 5.

Still, the final *cis* or *trans* conformations of the TFSI^−^ and DFTFSI^−^ anions in the crystal structure are subject to additional considerations that are not fully captured by isolated molecular models. For example, the ion-pairs are not screened in an isotropic medium, but rather a crystal field. Entropic contributions might also play an important role in the conformation of the anions within the crystal. Indeed, TFSI^−^ is found in both *cis* and *trans* conformations depending on the experimental temperature, indicating solid-solid phase transitions (Henderson et al., 2005; Herstedt et al., 2006; Zhou et al., 2011). While the latter is out of the scope of the work and is generally challenging for DFT methods, we addressed the former by constructing additional crystalline models for KTFSI and KDFTFSI, where the anions appear in both conformations. Figure 6 shows the relaxed crystal structures of KTFSI and KDFTFSI salts with *cis* and *trans* conforming anionic units. The calculations reveal that the crystal structures with *cis* conformations are 57.8 (52.1) kJ mol^−1^ more stable than the *trans* counterparts for KTFSI (KDFTFSI). While such large observed energy difference could be due to the fact that the *trans* crystal structures are optimized to a less stable local minimum, we argue that the preference of K^+^ for a tridentate K···O coordination in the isolated models, allow for a more efficient crystal packing with *cis* conforming anions. In fact, the original LiTFSI crystal structure reported (Nowinski et al., 1994) showed that each *trans* TFSI^−^ anion forms a bidentate Li···O coordination, such that each Li^+^ only interacts with the TFSI^−^ anions located along the crystallographic **a**-axis. Our calculations reveal that the structure undergoes significant distortions after substituting Li^+^ by the larger K^+^, where the latter tries to coordinate with other O ions along the **b**-axis (Figure 6), but this coordination is incomplete when compared to that of the *cis* structure.

The calculated structural descriptors of the isolated TFSI^−^, DFTFSI^−^, KTFSI, and KDFTFSI *trans* (*cis*) models are shown in Table 4. All cases show values that compare well with the experiments shown in Table 2. Additionally, DFTFSI^−^ shows H‒C distances of 1.095 (1.095) Å, and angles S‒C‒H 108.9 (109.2)°; F‒C‒H 110.6 (110.0)°. Our calculations show that very slight differences exist between *cis* and *trans* conformers in terms of bond distances and angles, both for TFSI^−^ and DFTFSI^−^ anions. The inclusion of K^+^ does not lead to significant differences in this regard either. However, noticeable differences can be seen in the C‒S···S‒C dihedral angles. In the case of TFSI^−^, we observe an angle of 173.3° and 39.4° for the *trans* and *cis* conformers, respectively, while the dihedral angles of DFTFSI^−^ are 149.7° and 28.8°. Indeed, these differences in the dihedral angles could be ascribed to the above-mentioned increased stability of the *trans* conformers of the DFTFSI^−^ anions, where an additional H···O interaction is observed. Thus, in DFTFSI^−^, the CFX groups do not present a fully eclipsed relative disposition in the minimum energy state of the *cis* conformer. In the case of potassium salts, KTFSI and KDFTFSI, *trans* (*cis*) dihedral angles are 173.4 (77.7)°, and 176.6 (78.7)°, respectively. The inclusion of the K^+^ cation results in larger dihedral angles of the *trans* conformers of KDFTFSI, while the contrary is true or KTFSI. The relaxation of the *trans* conformer of KDFTFSI results in a reconfiguration of the CF_2_H group, where the H···O interaction is no longer present (Figure 5D), which could explain the increment on the value of the dihedral angle. Looking at the *cis* conformer, the inclusion of the K^+^ cation results in larger dihedral angles for both KTFSI and KDFTFSI. Additionally, the structural descriptors of the *trans* (*cis*) models for the KTFSI and KDFTFSI crystals are shown in Table 5. Once again, all bond distances and angles correlate well with the experimental values shown in Table 2, with small differences between the *cis* and *trans* conformers. However, we can see that their bond distances are slightly overestimated probably due to the use of the PBE functional. The dihedral angles for KTFSI and KDFTFSI with *trans* (*cis*) configurations are 157.6 (17.8)°, and 149.3 (28.1)°, respectively. As we expected, these values are closer to experimental ones when compared to those of the isolated ion-pair calculations and show the importance of the crystalline environment in the geometry and conformation of molecular solids. Lastly, we note that the dihedral angles of the *cis* conformers are slightly larger for KDFTFSI than for KTFSI, in agreement with the experimental observations (Table 2).

**TABLE 4:** Average contact distances (Å) and angles for KDFTFSI and KTFSI isolated ion-pairs, and DFTFSI^−^ and TFSI^−^ anions, as obtained by DFT calculations.

Compound	KDFTFSI–trans (cis)	KTFSI– trans (cis)	DFTFSI^−^–trans (cis)	TFSI^−^–trans (cis)
N‒S (Å)	1.576 (1.576)	1.569 (1.576)	1.585 (1.576)	1.576 (1.576)
S‒O (Å)	1.429 (1.440)	1.432 (1.440)	1.432 (1.432)	1.432 (1.432)
S‒C (Å)	1.834 (1.840)	1.848 (1.848)	1.840 (1.848)	1.848 (1.848)
C‒F (Å)	1.341 (1.336)	1.325 (1.320)	1.345 (1.336)	1.336 (1.336)
S‒N‒S (°)	127.7 (119.6)	127.6 (119.3)	122.9 (127.2)	125.5 (126.6)
N‒S‒O (°)	112.9 (113.3)	113.0 (113.6)	112.1 (115.9)	112.8 (113.1)
N‒S‒C (°)	102.3 (100.4)	102.6 (101.8)	103.7 (101.6)	102.0 (101.6)
O‒S‒O (°)	118.0 (115.9)	118.2 (116.1)	118.6 (119.0)	119.1 (119.1)
O‒S‒C (°)	104.5 (106.0)	103.9 (105.4)	104.3 (104.0)	103.6 (103.6)
S‒C‒F (°)	109.3 (109.2)	110.1 (109.6)	110.2 (110.6)	110.9 (110.7)
F‒C‒F (°)	108.7 (109.1)	108.9 (109.3)	108.1 (107.8)	108.2 (108.2)

**TABLE 5:** Average contact distances (Å) and angles for KDFTFSI and KTFSI crystalline models as obtained by DFT calculations.

Compound	KDFTFSI–trans (cis)	KTFSI– trans (cis)
N‒S (Å)	1.592 (1.601)	1.591 (1.592)
S‒O (Å)	1.457 (1.457)	1.457 (1.460)
S‒C (Å)	1.880 (1.880)	1.880 (1.880)
C‒F (Å)	1.345 (1.352)	1.336 (1.336)
S‒N‒S (°)	128.7 (125.9)	129.3 (126.5)
N‒S‒O (°)	112.9 (111.8)	113.0 (111.8)
N‒S‒C (°)	101.0 (104.8)	101.5 (105.5)
O‒S‒O (°)	117.3 (116.8)	118.1 (117.2)
O‒S‒C (°)	105.3 (105.1)	104.6 (104.6)
S‒C‒F (°)	109.1 (109.5)	110.1 (109.6)
F‒C‒F (°)	109.0 (109.1)	109.1 (109.3)

## 4 Conclusion

In summary, the crystal structure and cation-anion interactions of the potassium salt with a hydrogen-containing sulfonimide anion, (difluoromethanesulfonyl)(trifluoromethanesulfonyl)imide {[N(SO_2_ CF_2_H)(SO_2_CF_3_)]^−^, DFTFSI^−^}, have been thoroughly examined, and comparatively studied with the conventional perfluorinated sulfonimide salt, potassium bis(trifluoromethanesulfonyl)imide {K[N(SO_2_CF_3_)_2_], KTFSI}. From the single-crystal X-ray diffraction analyses, the KDFTFSI salt shows a layered structure alternating cationic and anionic layers, in which K^+^ cations are sandwiched by the oxygen atoms in the sulfonyl groups. Despite a similar stacking pattern, KDFTFSI presents a higher bond valence for K···O contacts as compared to KTFSI, which is a clear sign for the increased cation-anion interactions by replacing highly electronegative fluorine with hydrogen atom in the former salt. The DFT calculations further reveal that the *cis* conformation is the most stable geometries for both TFSI^−^ and DFTFSI^−^ anions, especially when the effects of dielectric screening and interaction with K ions are accounted for in the calculations. Moreover, the presence of H-bonding in the DFTFSI^−^ anion brings a higher energy gap for the conversion between *trans* and *cis* conformers. The structural information of KDFTFSI provided in this work is of great benefit to getting a deeper understanding on the unique properties of the DFTFSI-based liquid and solid polymer electrolytes, thereby promoting the development of high-performance rechargeable batteries.

## Data Availability

The datasets presented in this study can be found in online repositories. The names of the repository and accession number(s) can be found below: https://www.ccdc.cam.ac.uk/, CCDC 2242830.
