# Supplementary material for: Crystal structure and cation-anion interactions of potassium (Difluoromethanesulfonyl) (trifluorome thanesulfonyl)imide
Source: Front Chem. 2023 Jul 12;11:1191394. doi: 10.3389/fchem.2023.1191394 (PMC10368979; doi:10.3389/fchem.2023.1191394)
Supplement: Supplementary file 1 [file DataSheet1.docx]

Supplementary Material

Crystal Structure and Cation-Anion Interactions of Potassium (Difluoromethanesulfonyl)(trifluoromethanesulfonyl)imide

Eduardo Sánchez-Diez^1*^, Lorena Garcia^1^, Oier Arcelus^1^, Lixin Qiao^1^, Leire San Felices^2^, Javier Carrasco^1,3^, Michel Armand^1^, Maria Martínez-Ibañez^1^, Heng Zhang^4*^

^1^Centre for Cooperative Research on Alternative Energies (CIC EnergiGUNE), Basque Research and Technology Alliance (BRTA), Vitoria-Gasteiz, Spain

^2^Servicios Generales de Investigación SGIker, Facultad de Ciencia y Tecnología, Universidad del País Vasco, UPV/EHU, Bilbao, Spain

^3^IKERBASQUE, Basque Foundation for Science, Plaza Euskadi 5, 48009 Bilbao, Spain

^4^Key Laboratory of Material Chemistry for Energy Conversion and Storage (Ministry of Education) School of Chemistry and Chemical Engineering Huazhong University of Science and Technology, Wuhan, China

*** Correspondence:**Eduardo Sánchez-Díez
esanchez@cicenergigune.com

Heng Zhang
hengzhang2020@hust.edu.cn

# Supplementary Data

**Crystallographic data**

**Table S1 Bonding parameters of KDFTFSI.**

| **Compound** | **KDFTFSI** |
| --- | --- |
| N‒S (Å) | 1.573(5), 1.581(6), 1.580(5), 1.562(6) |
| S‒O (Å) | 1.433(5), 1.435(5), 1.439(5), 1.435(5), 1.426(5), 1.436(5), .436(5),1.429(5) |
| S‒C (Å) | 1.807(8), 1.719(7), 1.726(7), 1.826(7) |
| C‒F (Å) | 1.325(9), 1.331(10), 1.351(10), 1.3889 (10), 1.3886(10), 1.391(10), 1.337(11), 1.293(10), 1.278(13), 1.3888(10), 1.3888(10), 1.3887(10) |
| S‒N‒S (°) | 128.0(4), 125.7(3) |
| N‒S‒O (°) | 116.5(3), 109.5(3), 106.4(3), 116.3(3), 106.8(3), 115.5(3), 117.0(3), 08.7(3) |
| N‒S‒C (°) | 104.4(3), 106.0(3), 105.9(3), 103.7(3) |
| O‒S‒O (°) | 116.1(3), 117.3(3), 117.2(3), 116.9(3) |
| O‒S‒C (°) | 105.0(3), 103.5(3), 104.8(4), 104.9(4), 105.0(3), 105.4(3), 105.4(3), 03.2(3) |
| F‒C‒F | 111.2(7), 98.8 (7), 108.4(6), 108.4(8), 107.6(8), 102.8(6), 104.0(6), 107.35(15), 108.01(12), 110.72(16) |
| S‒C‒F | 107.8(5), 110.2(6), 109.6(5), 114.3(4), 118.6(5), 113.2(5), 109.7(6), 110.6(6), 112.3(7), 111.6(4), 114.4(5), 116.0(5) |
| M···O (Å) avg. | 2.864(5) |
| M···O (Å) range | 2.687(5)‒3.338(6) |
| M···N (Å) | 3.295(6), 3.365(5) |
| C‒S···S‒C (\|°\|) | 17.1, 20.0 |

^[a]^ The data of KTFSI is obtained from (Xue et al., 2002).

| **Table S2 Fractional Atomic Coordinates (×10^4^) and Equivalent Isotropic Displacement Parameters (Å^2^×10^3^). U_eq_ is defined as 1/3 of of the trace of the orthogonalised U_IJ_ tensor.** | | | | |
| --- | --- | --- | --- | --- |
| **Atom** | ***x*** | ***y*** | ***z*** | **U(eq)** |
| K1 | 7540.5(8) | 1778.1(11) | 1679.1(12) | 35.5(4) |
| K2 | 7501.7(6) | 4376.3(9) | 3646.4(11) | 24.5(3) |
| S1A | 6541.8(7) | 1825.4(11) | 3830.6(13) | 23.8(4) |
| S1B | 6654.4(7) | 3067.3(11) | -369.8(12) | 21.2(4) |
| S2A | 6638.2(7) | 981.6(11) | 5896.9(13) | 24.4(4) |
| S2B | 6518.5(7) | 4728.2(11) | 985.0(13) | 23.7(4) |
| F1B | 5576(3) | 2314(5) | -79(5) | 75.4(19) |
| F4A | 5497(2) | 1392(5) | 6368(5) | 70.1(17) |
| F4B | 5623(3) | 5827(4) | 1655(5) | 65.6(16) |
| O1A | 6658(2) | 867(4) | 3350(4) | 30.6(10) |
| O1B | 6910(2) | 2094(3) | -193(4) | 32.8(11) |
| O2A | 6859(2) | 2657(4) | 3373(4) | 30.5(10) |
| O2B | 6926(2) | 3668(3) | -1195(4) | 30.2(11) |
| O3A | 6857(2) | 1397(4) | 6888(4) | 32.0(11) |
| O3B | 6576(2) | 5383(3) | 89(4) | 35.6(12) |
| O4A | 6944(2) | 105(3) | 5502(4) | 33.4(11) |
| O4B | 6862(2) | 4983(4) | 1926(4) | 38.7(12) |
| N1A | 6586(2) | 1879(4) | 5077(5) | 26.4(12) |
| N1B | 6593(2) | 3570(4) | 764(4) | 23.9(11) |
| C1A | 5738(3) | 2092(6) | 3539(6) | 36.4(16) |
| C1B | 5881(4) | 2834(8) | -822(7) | 48(2) |
| C2A | 5899(3) | 610(4) | 6159(5) | 55(2) |
| C2B | 5768(3) | 4843(3) | 1402(5) | 51(2) |
| F2B | 5884(3) | 2321(6) | -1699(6) | 71(2) |
| F3A | 5386(2) | 1321(5) | 3872(5) | 45.5(15) |
| F5A | 5788(4) | 35(9) | 7064(8) | 84(4) |
| F6B | 5323(3) | 4567(7) | 664(8) | 75(3) |
| F1A | 5679(2) | 2154(4) | 2485(4) | 51.1(12) |
| F2A | 5568(3) | 2940(5) | 4012(6) | 47.6(16) |
| F3B | 5584(3) | 3643(7) | -993(7) | 70(2) |
| F5B | 5633(4) | 4336(6) | 2344(6) | 96(3) |
| F6A | 5650(3) | 19(6) | 5355(5) | 82(2) |

| **Table S3 Anisotropic Displacement Parameters (Å^2^×10^3^). The Anisotropic displacement factor exponent takes the form: -2π^2^[h^2^a*^2^U_11_+2hka*b*U_12_+…].** | | | | | | |
| --- | --- | --- | --- | --- | --- | --- |
| **Atom** | **U_11_** | **U_22_** | **U_33_** | **U_23_** | **U_13_** | **U_12_** |
| K1 | 58.4(10) | 24.1(8) | 24.0(8) | 1.6(6) | 0.2(7) | 0.1(6) |
| K2 | 37.7(7) | 15.7(7) | 20.1(7) | -0.6(5) | 1.0(5) | 1.1(5) |
| S1A | 22.7(7) | 21.7(8) | 27.1(8) | 2.5(6) | -1.0(6) | 0.2(5) |
| S1B | 24.0(7) | 17.6(7) | 22.2(8) | 0.5(6) | 1.9(6) | 0.1(5) |
| S2A | 27.3(7) | 21.8(8) | 24.2(8) | 1.1(6) | -0.7(6) | -1.4(6) |
| S2B | 29.5(8) | 18.4(7) | 23.1(8) | -1.0(6) | 1.3(6) | 2.0(6) |
| F1B | 52(3) | 102(5) | 72(4) | -11(4) | 12(3) | -40(3) |
| F4A | 33(2) | 106(5) | 71(4) | -2(3) | 10(2) | 8(3) |
| F4B | 62(3) | 50(3) | 85(4) | -13(3) | 18(3) | 26(3) |
| O1A | 35(2) | 27(2) | 30(3) | -3(2) | -3(2) | 2.7(19) |
| O1B | 42(3) | 18(2) | 39(3) | 2(2) | 6(2) | 4.6(19) |
| O2A | 34(2) | 28(2) | 30(3) | 8(2) | 1(2) | -5.3(19) |
| O2B | 45(3) | 24(2) | 21(2) | 1.0(18) | 9(2) | 0(2) |
| O3A | 40(3) | 30(3) | 26(2) | -5(2) | -5(2) | 2(2) |
| O3B | 59(3) | 19(2) | 28(3) | 6(2) | 8(2) | 5(2) |
| O4A | 50(3) | 21(2) | 30(3) | -1.2(19) | 2(2) | 6(2) |
| O4B | 50(3) | 34(3) | 32(3) | -12(2) | -9(2) | 7(2) |
| N1A | 32(3) | 19(3) | 29(3) | -1(2) | -1(2) | -1(2) |
| N1B | 32(3) | 18(3) | 21(3) | 3(2) | 1(2) | 3(2) |
| C1A | 31(3) | 43(4) | 35(4) | 7(3) | -3(3) | 4(3) |
| C1B | 30(4) | 71(6) | 42(5) | -20(4) | -4(3) | -3(4) |
| C2A | 58(5) | 60(6) | 48(5) | 2(4) | 0(4) | -16(5) |
| C2B | 50(5) | 41(5) | 62(6) | -2(4) | 11(4) | 9(4) |
| F2B | 46(3) | 110(6) | 57(4) | -57(4) | -6(3) | -8(4) |
| F3A | 27(3) | 56(4) | 53(4) | 18(3) | -3(3) | -11(3) |
| F5A | 48(5) | 112(9) | 92(8) | 60(7) | -5(5) | -15(6) |
| F6B | 30(4) | 76(6) | 119(9) | -46(6) | -1(5) | 11(4) |
| F1A | 38(2) | 75(3) | 40(3) | 15(2) | -11(2) | 0(2) |
| F2A | 41(3) | 45(3) | 57(4) | 7(3) | 2(3) | 24(3) |
| F3B | 48(4) | 97(7) | 66(5) | -14(5) | -24(4) | 27(4) |
| F5B | 93(5) | 83(5) | 111(7) | 29(5) | 61(5) | 18(4) |
| F6A | 63(4) | 99(5) | 84(5) | -22(4) | -2(4) | -47(4) |

| **Table S4 Hydrogen Atom Coordinates (Å×10^4^) and Isotropic Displacement Parameters (Å^2^×10^3^).** | | | | |
| --- | --- | --- | --- | --- |
| **Atom** | ***x*** | ***y*** | ***z*** | **U(eq)** |
| H1A | 5710(170) | 2630(130) | 4000(200) | 101 |
| H1AA | 5580(30) | 1536(16) | 3840(20) | 101 |
| H1BA | 6010(30) | 2460(20) | -1380(30) | 101 |
| H1B | 5770(30) | 3483(12) | -860(30) | 101 |
| H2A | 5760(30) | 270(40) | 5580(30) | 101 |
| H2AA | 5940(30) | 240(20) | 6758(18) | 101 |
| H2BA | 5459(14) | 4574(19) | 1020(20) | 101 |
| H2B | 5730(20) | 4461(18) | 1993(16) | 101 |

| **Table S5 Atomic Occupancy for F and H atoms in KDFTFSI.** | | | | | | | |
| --- | --- | --- | --- | --- | --- | --- | --- |
| **Atom** | ***Occupancy*** |  | **Atom** | ***Occupancy*** |  | **Atom** | ***Occupancy*** |
| F2B | 0.8 |  | F3A | 0.75 |  | F5A | 0.6 |
| H1A | 0.25 |  | H1AA | 0.25 |  | H1BA | 0.23 |
| H1B | 0.27 |  | H2A | 0.2 |  | H2AA | 0.3 |
| H2BA | 0.29 |  | H2B | 0.21 |  | F6B | 0.62 |
| F2A | 0.75 |  | F3B | 0.7 |  | F5B | 0.88 |
| F6A | 0.9 |  |  |  |  |  |  |

**Table S6 Atoms and symmetry operators in KDFTFSI.**

| **Atom** | ***Symmetry*** |  | **Atom** | ***Symmetry*** |
| --- | --- | --- | --- | --- |
| K1 | x, y, z |  | K2 | x, y, z |
| N1A | 1.5-x, 0.5-y, -0.5+z |  | O1A | 1.5-x, 0.5+y, z |
| N1B | x, y, z |  | O1B | 1.5-x, 0.5-y, 0.5+z |
| O1A | x, y, z |  | O2A | x, y, z |
| O1B | x, y, z |  | O2B | x, 1-y, 0.5+z |
| O2A | x, y, z |  | O3A | 1.5-x, 0.5-y, -0.5+z |
| O2B | 1.5-x, 0.5-y, 0.5+z |  | O3B | x, 1-y, 0.5+z |
| O3A | 1.5-x, 0.5-y, -0.5+z |  | O4A | 1.5-x, 0.5+y, z |
| O3B | 1.5-x, -0.5+y, z |  | O4B | x, y, z |
| O4A | x, -y, -0.5+z |  |  |  |
| O4B | 1.5-x, -0.5+y, z |  |  |  |

**NMR data**

**Figure S1 ^1^H-NMR KDFTFSI**

**Figure S2 ^13^C-NMR KDFTFSI**

**Figure S3 ^19^F-NMR KDFTFSI**

**References**

Xue, L., Padgett, C. W., DesMarteau, D. D., & Pennington, W. T. (2002). Synthesis and structures of alkali metal salts of bis[(trifluoromethyl)sulfonyl]imide. *Solid State Sciences*, *4*(11), 1535–1545. doi: 10.1016/S1293-2558(02)00050-X
